# Instructions for External Focus of Attention Improved Taekwondo Kicking Performance Only Among Less Skilled Youth

**DOI:** 10.1177/00315125221083748

**Published:** 2022-04-09

**Authors:** Simo Siltanen, Reijo Bottas

**Affiliations:** Faculty of sport and health sciences, 541605 University of Jyväskylä, Jyvaskyla, Finland

**Keywords:** focus of attention, verbal instructions, taekwondo, side kick, performance

## Abstract

External focus of attention (EFA) studies among children have yielded more equivocal results than have those among adults. Some investigators have found an internal focus of attention (IFA) advantage in children and have explained their results by children’s generally lower skill levels, compared to adults. According to the constrained action hypothesis, children’s lower skill levels are not yet associated with over-learned automatic movement patterns, so their motor performance is not disrupted by IFA instructions. In this study, our objective was to examine a possible interaction effect between children’s skill levels and their exposure to either IFA or EFA instructions on motor performance. Our participants were 40 10–15-year-old taekwondo competitors of higher and lower skill levels (based on both the participant’s experience and their test performance) who engaged in a taekwondo kicking movement before and after either IFA or EFA instructions. We found improved kicking performance with *EFA* versus IFA instructions only among *less* versus more skilled participants.

## Introduction

Many studies have sought to identify the most fruitful direction of attentional focus (i.e., internal vs. external; Wulf, 2013) in its effect on motor performance (e.g., Abdollahipour et al., 2017; Abswoude et al., 2018; Halperin et al., 2017; McNamara et al., 2019). An internal focus of attention (IFA) involves a primary focus on a specific body part, such as the arm when punching, whereas an external focus of attention (EFA) refers to a primary focus on the movement target or outcome, such as when punching a pad (Halperin et al., 2017). Fewer studies have investigated movement tasks that simultaneously involve several skills (e.g., spatial accuracy and force or speed). As speed and accuracy negatively affect each other, the performer must find an ideal compromise between them (Elliott et al., 2004). While studies of attentional focus in combat sports remain scarce, Halperin et al. (2017) found EFA instructions to be superior to IFA instructions among expert and intermediate adult boxers. Several researchers have suggested that the constrained action hypothesis (CAH) (McNevin et al., 2003; Wulf & Prinz, 2001) best explains the common finding that EFA is superior to IFA (Abdollahipour et al., 2016; Ille et al., 2013; Wulf, 2013; Wulf et al., 2000) in that EFA enhances performance for movements that are more automatic and reflex-based (McNevin et al., 2003; Wulf & Prinz, 2001). Another theory advanced to explain the benefits of EFA has been the common coding theory (Prinz, 1997; Wulf, 2013), positing that perception and action planning have the same brain representation so that movements are more effective when they are planned, based on their intended outcome (Prinz, 1997; Wulf, 2013).

Wulf (2013) argued that the superiority of EFA over IFA instructions can be generalized to participants of all skill levels. But some studies have found that skilled participants benefit more from EFA versus IFA instructions, while less skilled participants benefit more from IFA versus EFA instructions (Chharbaghi et al., 2010; Perkins-Ceccato et al., 2003). Beilock et al. (2002) suggested that this is because a well-learned skill can be successfully implemented without attending heavily to its mechanics. When a well-learned skill becomes automated, conscious control over its execution may even be associated with an impaired performance, as this focus may limit the automaticity of the movement (McNevin et al., 2003; Wulf & Prinz, 2001). Some studies have found that learners who self-monitor the direction of their attention produce better results than either persistently imposed EFA or IFA (Miçooğulları et al., 2012).

Another potential mediating factor for optimal attentional focus in motor learning has been age (Roshandel et al., 2017). Research with children has supported the relative efficacy of EFA for such various motor tasks as gymnastics (Abdollahipour et al., 2015), basketball (Perreault & French, 2015) and tennis (Hadler et al., 2014). However, studies with children have produced even more contradictory results than studies with adults. Several child studies found no difference between the benefits of EFA and IFA instructions (Abswoude et al., 2018; Agar et al., 2016; Perreault & French, 2016). Both Emanuel et al. (2008) and Roshandel et al. (2017) directly compared groups of children and adults and concluded that adults benefitted more from EFA instructions, while children did not. Other studies also found IFA best for children (Tse, 2019). IFA advantages for children have been explained by children’s skill levels, as children are considered novices whose motor skills are not yet automated, meaning that IFA instructions do not disrupt automated movement, nor do IFA instructions limit motor skill performance (Roshandel et al., 2017). Abswoude et al. (2018) suggested that children would perform best by choosing their own focus, as they may benefit from both EFA and IFA guidelines during motor performance.

In this study, to better understand the influence of the skill factor in determining the optimal focus of attention for children’s motor performance, we compared young competitors of varied skill levels who were exposed to EFA and IFA instructions on a taekwondo side kick performance, involving both accuracy and force production. Based on the constrained action hypotheses, we expected EFA (but not IFA) instructions to improved impact forces of higher skilled participants, while IFA (but not EFA) instructions would improve impact forces of lower skilled participants.

## Method

### Participants

This study utilized data originally collected as part of routine coaching purposes. 40 young (10–15-year-old; *M* age = 12.6 years) taekwondo competitors volunteered for this study (Table 1). Following our explanations of the study, each participant gave their assent, and parents or legal guardians of all participants gave their written informed consent to use previously gathered kicking data in our analyses. The study was conducted according to the principles of the declaration of Helsinki. According to guidelines of the Finnish National Board on Research Integrity, provided to us by the chair of our ethics committee on this journal editor’s inquiry, our research did not require prior ethical review because (a) we obtained voluntary child assent and separate voluntary informed parental consent for participation; (b) our research did not involve intervening in the physical integrity of research participants; (c) our study did not expose our participants to strong stimuli or mental harm; and (d) and our study presented no threat to the safety of participants or family members closest to them.

**Table 1.** Participant Data

**Group**	**Group size**	**Age Mean (SD)**	**Weight category (median + range)**
**Beginner level**	*n* = 14 (4 females)	11.9 (1.5)	Under 37 kg (from −29 kg to −65 kg)
**National level**	*n* = 19 (6 females)	12.7 (1.6)	Under 41 kg (from −29 kg to −68 kg)
**International level**	*n* = 7 (3 females)	13.9 (1.1)	Under 51 kg (from −37 kg to −68 kg)
**Lower performance**	*n* = 21 (7 females)	12.1 (1.6)	Under 37 kg (from −29 kg to −65 kg)
**Higher performance**	*n* = 19 (6 females)	13.2 (1.5)	Under 44 kg (from −33 kg to −68 kg)

### Procedure

This experiment was co-organized with the national taekwondo federation at two separate locations. Most of the experiment was conducted during The National Taekwondo Training Center’s camp, and other tests were completed in the dojang (training facility) of the Taekwondo Club. Testing conditions were similar in both locations. Neither setting was a laboratory site, but each setting had a similar mattress on the floor and a quiet space at the side of the dojang.

The studied task was the front leg side kick, and all the equipment used in the test was familiar to all participants before the test. All participants completed a 10-minute warm-up before the experiment. In the warm-up, they practiced side kicking and confirmed their readiness to participate. At the experimental site, we collected background information from the participants, showed them how the testing equipment functioned and informed them about the experimental task.

**Figure 1.**
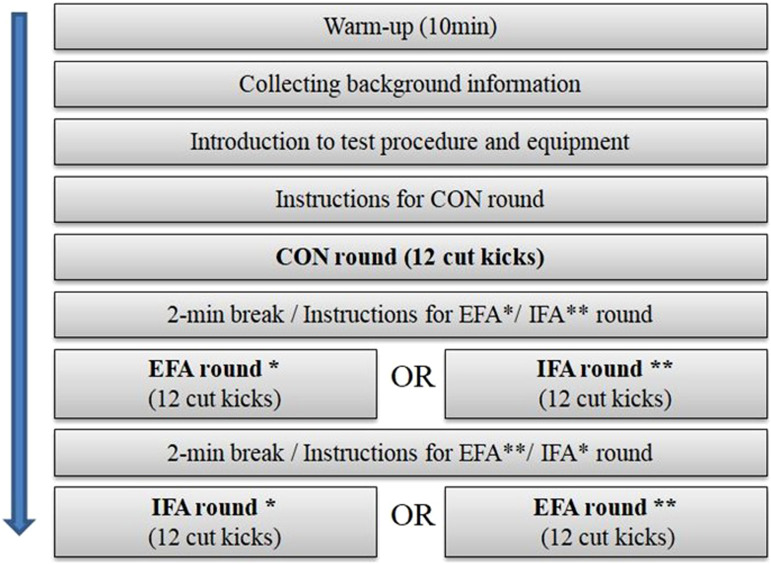
Test Procedure. After the first control round, the external focus of attention and internal focus of attention rounds were conducted in reverse order with every other participant. * shows the procedure for participants with odd participant numbers (*n* = 20) and ** the procedure for participants with even numbers (*n* = 20).

The task consisted of three rounds of kicks (Figure 1)—a first control round (CON) and then two consecutive testing rounds in which the participants received instructions regarding direction of their focused attention (IFA= internal focus of attention and EFA= external focus of attention). We divided all participants into two equally sized groups that received two different testing orders: (a) CON → EFA → IFA (*n*=20; *M* age = 12.6 years*, SD* = 1.66) and CON → IFA → EFA (*n*=20; *M* age =12.6 years*,* SD = 1.085). In each round, participants executed 12 front leg cut kicks at the target, using each leg six times.

First, in the control round (CON), participants received the following instructions: “Your task is to kick the target 12 times as powerfully and accurately as possible by using the front leg cut kick. Use each leg six times, choose whether to start kicking with the right or left leg, but do the first six kicks using the same leg. On the bottom of your feet, you can see two sensors. These have to hit the target for the kick to be registered by the system. You will be given a signal from the researcher before each kick. There is no hurry to finish the test, so concentrate fully on each kick.” While instructing the participant, a researcher demonstrated the technique to be used in the experiment and where to strike the target. The researcher also pressed the sensors on the bottom of the participant’s feet to show what part of the foot had to hit the target. No instruction was given on kicking distance; and, thus, participants were free to use the distance that best suited them.

In the second and third rounds, participants continued with the same task; but, for these rounds, participants were given either IFA or EFA instructions on how to focus their attention during kicking. The internal versus external instructions order reversed for every other participant. The instruction for the internal focus of attention (IFA) was as follows: “Focus on the movement of your kicking leg. Try to extend your leg as powerfully and accurately towards the target as possible.” The instruction for the external focus of attention (EFA) was as follows: “Focus on the target. Try to kick the target as powerfully and accurately as possible.” The participant first read the instructions from a note, after which the researcher highlighted the main points of the IFA, demonstrating the movement and telling the participant to focus on extending the leg powerfully. In giving the instruction for the external focus of attention (EFA), the researcher highlighted the target by showing it and instructed the participant to focus on hitting it as powerfully as possible. Before starting the round, the participant had to answer the question, “Where do you have to aim your focus during the next round?” The purpose was to make sure the participant understood the instructions. The participant received no feedback on their performance during the experiment. In each round, after six kicks, the researcher reminded the participant where to focus. Participants were allowed a two-minute break between rounds for resting, during which they were given no stimulus other than the instructions at the end of the break for the next round of kicks.

### Materials

We used the Daedo Gen2 electronic scoring system in this experiment. It is one of the official scoring systems used by World Taekwondo in international taekwondo tournaments, including the 2016 Rio de Janeiro Olympics. The experimental equipment included a size 3 electronic body protector, Gen2 electronic foot gear and TK-strike software for the computer. A body protector was fitted to a body opponent bag (BOB) attached to a stand filled with sand and water. The body protector size #3 was determined to be one best fit to the BOB. The height of the kicking target was adjustable according to participants height. We registered and stored information about the strength of each kick on a computer using TK-strike software. The computer screen was turned away from the participant to prevent access to feedback on performance. We used the same equipment for all participants, except for the different sized foot gear needed by each participant.

The impact force (Daedo units) of every kick was measured by the foot gear sensors striking the electronic body protector, and this was shown on the computer screen and recorded for analysis. If the kick did not hit the foot gear sensors correctly, the impact force was smaller, and sometimes even recorded as zero. Thus, in addition to force, the task also required adequate accuracy. Accuracy was not measured separately, because it could only have been shown as a binary (i.e., present or absent) value. We defined the points scored as successful techniques in relation to the impact force hit levels of one’s own competition category (age, gender, weight class) (Daedo Truescore USA, 2021). We then defined the participant’s performance level, based on their CON round mean score.

### Statistical Analysis

In the data analysis phase, we divided participants into skill level groups in two ways and separately analyzed the relationship between skill level and attentional focus in each group. First, skill level and analyses were based on test performance as determined by the participants’ mean scored points performing the taekwondo kick during the CON round (i.e., participants who scored higher than the group median score (*n* =19) and participants who scored lower than the group median score (*n* = 21). Second, we based skill level and analyses on the participants’ prior experience (i.e., beginner level (*n* = 14), national level (*n* = 19) and international level (*n* = 7). We used Microsoft Excel for office 365 software for calculating CON rounds averages and medians for the divisions to performance groups.

We used the IBM Statistical Package for the Social Sciences (SPSS, v. 24, IBM Corp., New York) software for inferential statistical analysis to determine group differences. We analyzed the data on impact forces of the different rounds (CON/EFA/IFA) with repeated measures analysis of variance (ANOVA), and we used a Bonferroni test for pair-wise comparison. We checked the equality of variances (homogeneity of variance of higher performance & lower performance groups) for the impact force. For impact forces, all kicks (*N*=1440) were counted. We used the interactive dotplot tool for creating dot plot charts. We set statistical significance at *p* < .05.

## Results

### Skill Level: Test Performance

**Figure 2.**
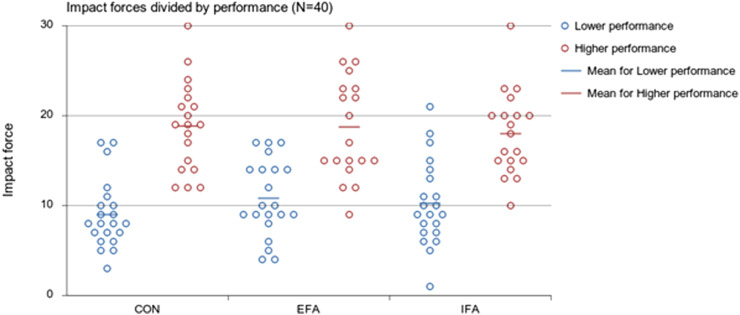
Side Kick Performance of Lower Performing Participants (*n* = 21) and Higher Performing Participants (*n* = 19). Note: The dot represents participants’ mean round score in Daedo units. The lower performance group improved their performance from first control round to external focus of attention significantly (*p* = .042).

Descriptively, CON round scores for the lower performance group were *M* = 8.9, *SD* = 3.8, and CON round scores for the higher performance group were *M* = 18.9, *SD* = 4.9 (see Figure 2). With EFA instructions, the lower performance group improved their side kick performance significantly as compared to their CON round (EFA *M* = 10.8, *SD* = 4.3; F (1,0)= 4.427, *p*=.042) but, with IFA instructions, the lower performance group did not perform differently than during their CON rounds. For the higher performance group, there were no differences between CON rounds during rounds with either EFA or IFA instructions.

### Skill Level: Experience

**Figure 3.**
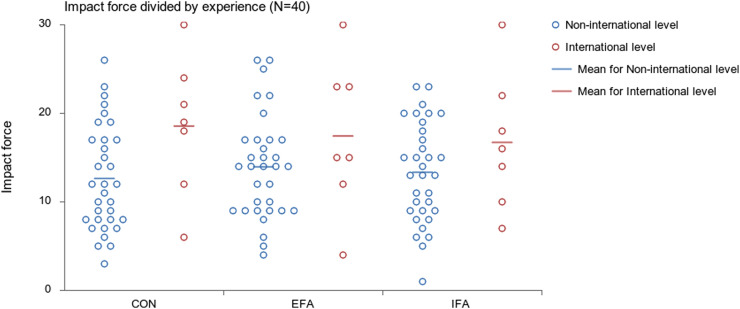
Side Kick Performance of Non-international Level Participants (*n* = 33) and International Level Participants (*n* = 7). Note: The dot represents participants’ mean round scores in Daedo units. Non-International level participant’s group improved their performance from first control round to external focus of attention round significantly (*p* = .018).

Descriptively, the participants’ experience-based CON round scores across experience level were as follows: (a) Beginner level *M* =10.0, *SD* = 5.2; (b) national level *M =*14.5, *SD* = 5.8; and (c) international level: *M =*18.6, *SD =* 7.8. Grouping non-international level (beginner and national) participants, their combined average during the CON round was *M* =12.6 (*SD* = 5.9; see Figure 3). While there were no significant differences in the CON rounds and EFA or IFA rounds of international level participants (see Figure 3), the combined non-international level participants (i.e., beginner and national level) showed a significant difference in their EFA rounds compared to their CON rounds (EFA *M* = 13.9, *SD* = 5.9; F (2,958) = 4.048, *p*=.018).

## Discussion

The purpose of this study was to examine how the skill levels of young taekwondo competitors interrelated with specific attention focus instructions on a motor performance test. We hypothesized that EFA, but not IFA, instructions would lead to improved performance among higher skilled participants, whereas, for less skilled participants IFA, but not EFA, instructions would lead to improved performance. Contrary to these hypotheses, participants with *lesser* skill levels (as defined by either lower performance scores or less experience) improved kicking performance, relative to their CON rounds, with *EFA*, but not with IFA, instructions.

These results were the opposite of our hypotheses and they were inconsistent with previous theory. In our study, the group that benefitted most from EFA instructions were lower performing and non-international level participants, while higher performing or international level participants did not improve their performance significantly with either EFA or IFA instructions. Perhaps the most skilled or experienced competitors found that either EFA or IFA instructions interfered with their personal preferences for focusing attention. Previously Stoate andWulf (2011) reported that IFA instructions were a hindrance to expert swimmers, compared to a control round in which there were no attentional focus instructions of any kind. Miçooğulları et al. (2012) studied the effects of direction of attentional focus on learning the “head kick” in soccer and found that personal control over the type of attentional focus (external or internal) had a greater effect on motor skill learning than either type of instructional focus applied across all participants. Thus, while EFA guidelines may generally produce better results than IFA guidelines in advanced performers, neither method may be as beneficial as personal control over attentional focus.

In this study, our interest was in the participants’ immediate performance. Thus, our results may have practical value to coaches for giving real time instructions to athletes during competitions and practice. We involved child competitors in this study because the fundamentals for competing in taekwondo are often established in childhood. Our study also differed from previous attentional focus studies conducted with children in that our experimental task primarily involved power but also required at least adequate accuracy; previous studies with children mainly focused on accuracy alone (Agar et al., 2016; Perreault & French, 2015, 2016).

### Strengths, Limitations, and Directions for Further Research

To obtain an adequate participant sample size, we conducted this study in field conditions with largely identical test venues. We measured our participants’ kicking performance with the Daedo Gen2 electronic system, since this system is used in official taekwondo competitions such as the Olympic Games. All our participants were familiar with the Daedo system. A strength of this study was our effort to ensure that participants understood the instructions given to them. In most previous studies, attentional focus instructions were only given verbally and demonstrated, without checking the participant’s understanding (Abdollahipour et al., 2015; Abswoude et al., 2018). For future research, it would also be interesting to ask participants after their performance, what they actually focused on during the test (or what focus they believe would be most helpful to success). A common limitation of many attentional focus studies, including this one, is an exclusive focus on measurable task outcomes (Hadler et al., 2014; Halperin et al., 2017) and judgments of any advantage to EFA instructions. A more nuanced outcome possibility in this study, for example, is that EFA instructions might have most directly affected higher impact forces, while IFA instructions might have most directly affected other kicking benefits that were not assessed.

## Conclusions

Attentional focus studies in children and novice adults have generally yielded equivocal results with respect to the instructional benefits of EFA; and most studies of participants at different skill levels and most theories to explain results have been based on adult research. Some contradictory findings regarding focus of attention in children’s learning and performance have been attributed to children’s relatively low levels of skill and movement automation, meaning that the constrained action hypothesis might give an advance to IFA over EFA instructions for most children and, particularly, for unskilled children.

The present study was the first to explore the relationship between children’s skill levels, based on their experience and testing performance, and the effects on performance of directing their attentional focus. Our results challenge the existing view of how skill level interacts with EFA instruction. We found EFA guidance to be effective for *less skilled* youth learning taekwondo, while more advanced participants failed to benefit significantly from either EFA or IFA instructions. More research on the association between attentional focus instructions and performance, especially in children, is needed. Based on research to date, generalizations about the benefits of instructional direction (internal vs. external) are harder to make for children than for adults (Miçooğulları et al., 2012; Saemi et al., 2013). It seems likely that factors other than direction of attentional focus are more important determinants of motor performance among children. Future research might focus on their discovery.
